# Uncovering Discrete Synaptic Proteomes to Understand Neurological Disorders

**DOI:** 10.3390/proteomes6030030

**Published:** 2018-07-19

**Authors:** Yi-Zhi Wang, Jeffrey N. Savas

**Affiliations:** Department of Neurology, Northwestern University Feinberg School of Medicine, Chicago, IL 60611, USA; yi-zhi.wang@northwestern.edu

**Keywords:** proteomics, basal ganglia, synapses, synapse specificity, neuronal circuits, axons, dendrites, neurodegeneration

## Abstract

The mammalian nervous system is an immensely heterogeneous organ composed of a diverse collection of neuronal types that interconnect in complex patterns. Synapses are highly specialized neuronal cell-cell junctions with common and distinct functional characteristics that are governed by their protein composition or synaptic proteomes. Even a single neuron can possess a wide-range of different synapse types and each synapse contains hundreds or even thousands of proteins. Many neurological disorders and diseases are caused by synaptic dysfunction within discrete neuronal populations. Mass spectrometry (MS)-based proteomic analysis has emerged as a powerful strategy to characterize synaptic proteomes and potentially identify disease driving synaptic alterations. However, most traditional synaptic proteomic analyses have been limited by molecular averaging of proteins from multiple types of neurons and synapses. Recently, several new strategies have emerged to tackle the ‘averaging problem’. In this review, we summarize recent advancements in our ability to characterize neuron-type specific and synapse-type specific proteomes and discuss strengths and limitations of these emerging analysis strategies.

## 1. Introduction

The mammalian nervous system is a complex organ assembled from millions of neurons in complex circuits arranged into elaborate networks. Neuronal networks provide information-processing capabilities and also facilitate the transfer of large amounts of data between brain regions and the rest of the organism [1]. To establish proper neuronal circuits during development, axons must innervate the appropriate target regions and form precise connections with the proper neurons [2]. Axons form short-range neuronal connections with nearby neuronal dendrites within the same brain region and long-range nerve fibers connect distant territories of the brain [3]. Chemical synapses represent specialized cell junctions that allow information, in the form of neurotransmitters, to flow from presynaptic axons to postsynaptic dendrites. Synaptic proteomes mature during development and are refined through activity dependent changes [4]. Proper synaptic communication is essential for many physiological functions from breathing to learning and memory [5,6].

The disruption of synaptic transmission from long-range projection neurons plays a key role in a variety of neurological disorders. For example, impaired neurotransmission of projection neurons is a defining characteristic of neurodegeneration in basal ganglia diseases including Parkinson’s disease (PD), Huntington’s disease (HD), and dystonia [7]. Death of substantia nigra dopaminergic neurons in PD patients leads to loss of a major afferent basal ganglia projection. Importantly, administration of levodopa mimicking dopamine is a widely used therapeutic for PD [8]. Furthermore, in HD, discrete projections are (i.e., indirect pathway) impaired and responsible for the core aspects of pathology [9]. Neurodevelopmental disorders including autism and schizophrenia are also believed to be caused by the impairment of multiple neuronal circuits [10,11]. Specific brain regions, neurons, synapses, and synaptic proteins are also impaired in Alzheimer’s disease (AD) [12,13]. Hippocampal synapses are the first to be affected in AD, but later pathology spreads to cortical regions and beyond. However, some brain regions such as the cerebellum seem to be resistant to the AD-related synaptic dysfunction even in late stages of AD [14]. Therefore, a deep understanding of the mechanisms and proteins regulating discrete synapses formed locally or between long-range axonal projections and their postsynaptic counterparts is a key step towards developing effective treatments for a wide range of neurological disorders.

Systematic characterization of neuron-neuron connections or the “connectome” has recently received significant biomedical research attention, since altered connections may play a causative role in neurological dysfunction. The goal is to map the complete set of anatomical and functional connectivity within the healthy mammalian brain. This is no small challenge since there are billions of neurons and 10^12–14^ synapses in the mammalian brain [15,16]. Clearly, this is an immense challenge that will require decades of research to complete [17]. While careful anatomical and electrophysiological measurements should eventually provide an invaluable description of how neuronal networks function, it is unlikely to facilitate the treatment of neurological disorders on its own, therefore necessitating a molecular understanding. Determining the proteins and molecular mechanisms responsible for altered circuit function of specific neurons and discrete synapses in discovery-mode, represents an important opportunity that is only now becoming achievable [18].

Technological improvements in mass spectrometry (MS)-based proteomic analysis in combination with the recent development of several chemical strategies, have now made it possible to probe discrete neuronal and synaptic proteomes in vitro and in vivo. In this review article, we summarize recent success in this emerging area of neuroproteomic research and provide a preview of how we and others are combining these new tools to increase our understanding of underappreciated synaptic mechanisms. The hope is that by identifying small groups of altered proteins in specific neurons and synapses, we will be able to advance our understanding of synaptic dysfunction and discover new therapeutic targets.

## 2. The Synaptic Protein Averaging Problem

Historically, neurons have been classified based on the identity of the neurotransmitter (NT) that their axons secrete. For example, glutamate is released in synaptic vesicles by glutamatergic neurons and plays a key role in learning and memory as a major excitatory NT type in the brain [19]. Other major NTs, including adrenalin, noradrenaline, dopamine, gamma aminobutyric acid (GABA), acetylcholine, and serotonin, regulate discrete functional aspects of neuronal physiology and manage different physiological aspects such as mood, pleasure, and reward. Neurotransmission is a complex and intricate neurobiological process and we already know the identity of more than 100 NTs, with many more still likely to be discovered. It is expected that the machinery responsible for the release of synaptic vesicles containing different NTs require at least some of the same core proteins. However, this has yet to be deeply investigated and it is possible that the release of different NTs requires a unique set of protein factors [4]. Furthermore, it is well known that co-release or co-transmission in the same or distinct synaptic vesicles is also a common mechanism in the mammalian brain [20]. For NTs to function, they must each bind their cognate postsynaptic receptor and facilitate signaling as ligand-gated ion channels, transporters and G-protein coupled receptors (GPCRs). This high degree of fidelity guarantees the faithful incorporation of multiple signals and minimizes unwanted NT crosstalk.

Individual neurotransmitters bind to multiple receptor proteins and protein complexes. Take metabotropic glutamate receptors (mGluRs) as an example, there are eight different GPCRs that localize to both pre- and post-synaptic membranes. The expression of mGluR4 is very high in the cerebellum but mGluR6 is highly expressed only in the retina [21]. The spatially restricted expression pattern of these receptors suggests that synapses contain a unique array of proteins in both the cerebellum and retina. Let us consider the basal ganglia, situated at the base of the forebrain, as an illustration of regional synaptic heterogeneity. It is composed of the striatum, globus palladus, ventral pallidum, substantia nigra, and subthalamic nucleus and is associated with a variety of functions [22]. The basal ganglia possess two groups of spiny projection neurons (SPNs), direct pathway (dSPNs) and indirect pathway SPNs (iSPNs). Interestingly, dSPNs and iSPNs have nearly indistinguishable morphology and are both GABAergic, however they control contrasting aspects of motor control. Both types of SPNs receive glutamatergic input from the cortex and thalamus, and dopaminergic projections from substantia nigra. Dopamine receptor D1 (Drd1) is selectively expressed in dendritic spines of dSPNs, Drd2 is selectively expressed in iSPNs, and these receptors have distinct signaling activities [23]. Previous targeted biochemical studies and translation profiling experiments have confirmed significant difference in dSPNs and iSPNs expression profiles, however the synaptic proteomes of these neurons has yet to be effectively compared [24]. Taken altogether, the synaptic protein composition of dSPNs and iSPNs are likely to be different in at least a few key aspects. Interestingly, dSPN and iSPN have distinct roles in neurodegenerative diseases and disorders such as PD and schizophrenia. The most effective medicines for these diseases have targeted Drd2 SPNs, suggesting the malfunction of Drd2 synapses play a key role [25]. Moreover, a recent study showed that neuroligin-3 (Nlgn3) mutations selectively impaired dSPNs in nucleus accumbens (NAc) to boost autism spectrum disorder (ASD)-associated repetitive behaviors, suggesting the malfunction of Nlgn3 positive synapses in NAc dSPNs may be a driver of at least some behavioral symptoms in ASD [26].

**Figure 1:**
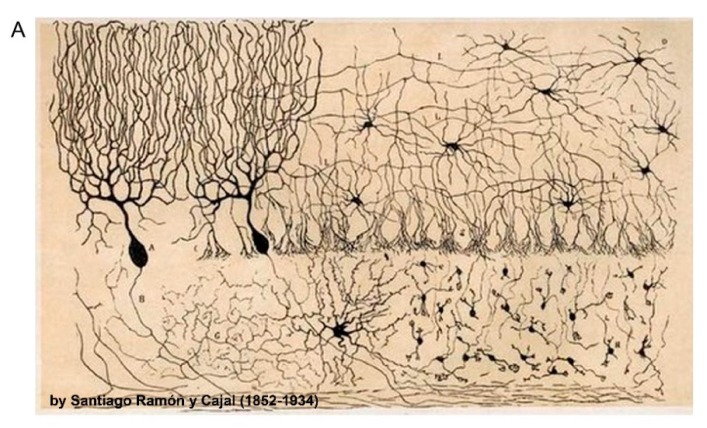

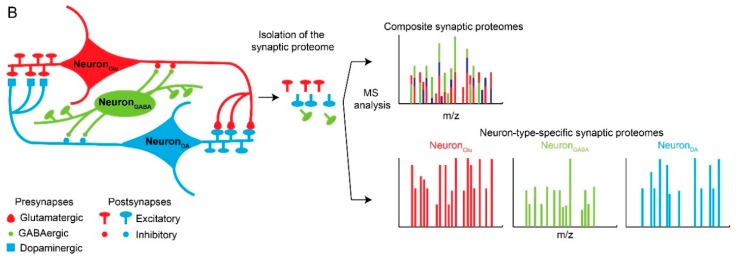
Neuronal and synaptic diversity complicates the interpretation of proteomic datasets. (**A**) Original drawing by Santiago Ramon Y Cajal showing a diverse neuronal population in chicken brain. Reproduced here without restriction since this work is in the public domain in its country of origin and other countries and areas where the copyright term is the author’s life plus 70 years or less. (**B**) Traditional biochemical approaches with mass spectrometry (MS) analysis of pre- and postsynaptic proteomes are limited by the molecular averaging (top). Analysis of discrete synaptic proteomes represents a major advancement in our ability to understand synapse specific functions.

The high degree of neuronal and synaptic heterogeneity in the brain poses a prohibitive obstacle for researchers trying to determine the protein composition of discrete synapses. Historically, the most widely used strategy has been to dissect individual brain regions, homogenize the tissue in sucrose buffer, perform differential centrifugation to purify synaptosomes, and use proteomics to identify the proteins present in the purified material. However, during the process of homogenization, the identity of all projection specific synaptic proteins will be lost and the resulting datasets will represent a composite ‘average’ measurement of synaptic protein content (Figure 1). Simply put, low abundance proteins present at all SPN synapses will be indistinguishable from those present at moderate levels only at iSPN or dSPN synapses. This is important since discrete neural circuits are affected in neurological disorders and by using the traditional synaptosome approach to evaluate altered synapses is suboptimal at best and misleading at worst. Thus, we need new approaches to facilitate in vivo proteomic characterization of discrete synapses in the context of rodent models of neurological disorders.

## 3. Strategies to Overcome Averaging at the Cellular Level

The accuracy and sensitivity of nearly all proteomic analysis workflows are limited by the degree of proteome complexity present in the extract. For example, the presence of even a few highly abundant synaptic proteins, such as CaMKII, tends to limit our ability to detect and quantify low abundant synaptic proteins in both targeted and untargeted MS-based analysis workflows [27]. The proteome complexity of synaptosomes prepared from rodent whole brain extracts poses an even greater challenge since they represent cumulative composite collections of proteins. Therefore, one commonly used strategy to surmount the averaging problem is to limit the heterogeneity of the protein extract [28]. With this goal in mind, multiple approaches have been developed and applied to the investigation of cell type and region-specific synaptic proteomes.

### 3.1. Laser Capture Microdissection (LCM)

**Figure 2:**
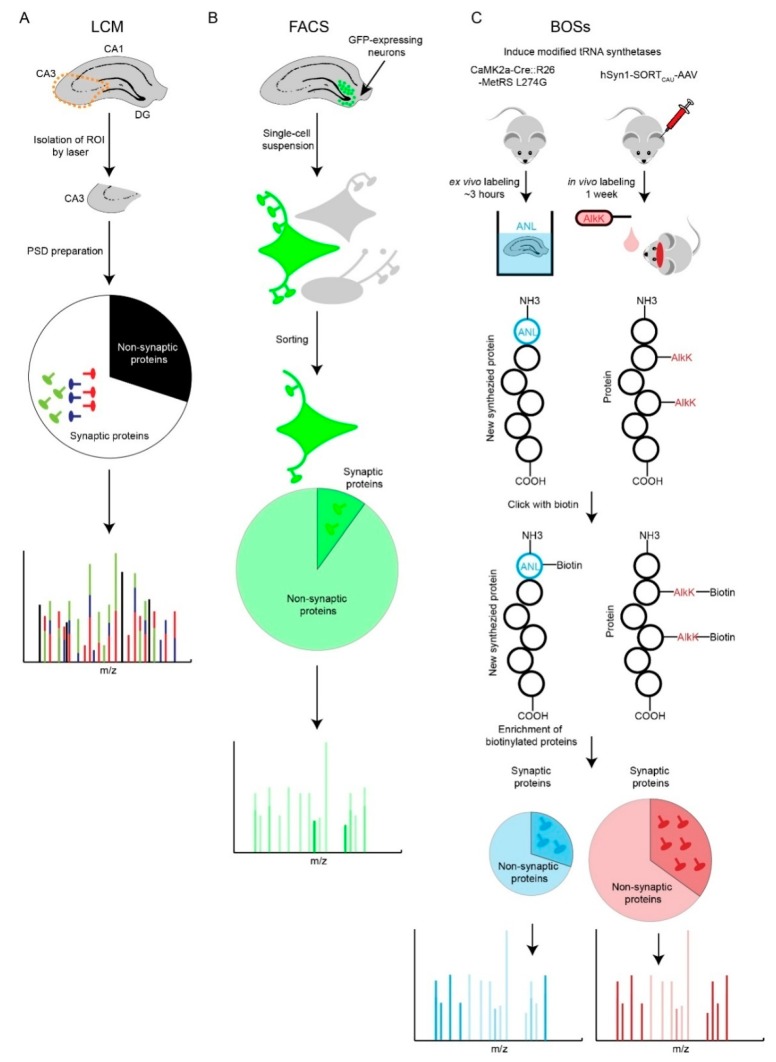
Comparison of Laser Capture Microdissection (LCM), Fluorescence-Activated Cell Sorting (FACS) and Bioorthogonal Strategies (BOSs) strategies. (**A**) LCM-based synaptic proteomic analysis is limited by potentially severe molecular averaging. (**B**) FACS-based proteomics is able to profile a specific type of neuron. However, loss of neurites and synapses during preparation of single-cell suspension is a shortfall. Most of the identified proteins are non-synaptic cytosolic proteins that localize to the soma. (**C**) Two BOSs-based proteomic strategies provide solutions for ‘averaging problem’ and potentially retain more synaptic information. AlkK is an economic alternative of CypK, which is affordable to feed rodents. Both BOSs can be used for ex vivo and in vivo labeling of cell-type specific proteins; due to space constraints, we illustrate one labeling strategy for each BOS.

LCM is a straightforward strategy to identify a tissue region of interest with a microscope and a laser to isolate it from intact brain tissue from any source for subsequent molecular characterization (Figure 2A). Typically, the brain tissue sections (5–50 micrometers thick) are mounted and examined with a light microscope. Regions of interest (i.e., certain group of neurons) are identified based on location or morphology, and targeted for isolation. Then the selected targets, which represent groups of cells or even a single cell, is precisely cut away from the brain section with an ultraviolet laser beam (usually 355 nm) and captured with an infrared laser [29]. LCM has high spatial resolution that can be precisely controlled within a few μms. Thus, the laser will only minimally damage the tissues adjacent to that of interest during the isolation and intact cellular structures such as synapses can be preserved. LCM has been widely used to study specific proteomes in discrete brain regions with laminar organization such as the hippocampus [30]. However, LCM has several significant limitations; it requires specific morphological characteristics and neuronal organization to identify synaptic regions of interest, time required to isolate the target tissue can compromise its integrity, and the depth of proteomic analysis is nearly always sample limited.

### 3.2. Fluorescence-Activated Cell Sorting (FACS)

FACS is the most commonly used technology to isolate discrete populations of dissociated cells of interest in a stream of fluid with an electronic detector [31]. FACS requires fluorescently labeled cells via dyes, antibodies, or fluorescent proteins [32]. Several fluorescent labels can be used in parallel and the cells can be sorted and collected based on the combination of fluorescence signals. The advantage of FACS is that, with appropriate labeling, multiple cell types of interest can be confidently isolated at the same time. However, FACS may not be an optimal strategy to isolate intact neurons, since the cells need to be individually dissociated into a single-cell suspension (Figure 2B). During this process, synapses and neurites may become damaged or retract. To overcome this challenge, researchers have recently used FACS to sort fluorescence-labeled purified synaptosomes. This approach can be used to isolate synaptosomes from discrete synapse-types (e.g., VGluT1-GFP) and in combination with MS, begin to determine synapse type specific proteomes [33]. This strategy addresses the synapse-loss and specificity problems but may be somewhat hindered by the ability of FACS to sort small heterogeneous vesicles (i.e., synaptosomes) with weaker fluorescence signals and the potential for clumping of fluorescent and non-fluorescent particles. Thus, neuronal debris and mitochondria may co-purify with fluorescent synaptosomes, and potentially lead to co-purification of non-synaptic proteins and potentially limit the specificity of this strategy.

### 3.3. Bioorthogonal Strategies (BOSs)

Recently, genetically modified tRNA synthetases in combination with unnatural amino acids and BOS have facilitated the identification and measurement of cell type specific proteomes [34,35]. Alvarez-Castelao et al. developed a methionyl-tRNA synthetase (MetRS) L274G-based system; MetRS L274G primes methionine tRNAs with the methionine amino acid surrogate azidonorleucine (ANL). When MetRS L274G is selectively expressed in discrete cells with cell type-specific promoters or Cre recombinase, ANL is selectively incorporated into nascent polypeptides, which can be clicked with biotin and isolated with streptavidin. Krogager et al. used pyrrolysyl-tRNA synthetase—tRNAXXX with FLEx-adeno-associated virus (AAVs) and Cre recombinase strategy to measure neuron-type specific proteomes [34]. Pyrrolysyl-tRNA synthetase-–tRNAXXX pair was only expressed in Cre-positive cells. In these cells, a non-canonical amino acid substrate with bioorthogonal cyclopropene group (CypK), leads to stochastic but low level labeling of the proteome with CypK. Proteins containing the non-canonical amino acids are clicked with biotin, and enriched with streptavidin. By combining BOS strategies with stable isotope-based quantitative proteomics, the measurement of discrete neuron-type specific proteomes in vivo can be achieved. BOSs are well suited to study cell type-specifically proteomes in vivo and represent an emerging field of chemical biology (Figure 2C). However, it is possible that ANL and CypK labeling impairs protein function. BOSs are dependent on new protein synthesis. It makes this strategy biased for those proteins with high translation rates and potentially slow degradation rates. Therefore, BOSs are suitable to probe neuron-type specific synaptic dynamic changes such as developmental maturation and activity-induced changes. Therefore, the measurement of long-live synaptic proteins will be in accessible [36].

## 4. Strategies to Overcome Averaging at the Molecular Level

Determining the proteome of a single type of neuron or synapse within the mammalian brain is a monumental challenge. Except for LCM, the strategies described above are not sufficiently sensitive or robust to identify and measure cell type-specific synaptic proteomes. To home in on synaptic proteins from the proteomic datasets generated from these approaches, it is common to filter protein lists based on publically available synaptic proteins databases, such as SynaptomeDB or G2C:Synapse Proteomics [37,38]. Overall, this strategy works well, however the confidence of one’s findings will only be as specific as the databases used to filter the datasets are accurate. For example, this strategy may be misleading since these proteins may not be present at synapses in the specific neurons under investigation. FACS and BOS-based approaches are powerful but may be hindered by two limitations when used to measure cell type-specific synaptic proteomes. The first limitation is that these strategies will provide analyses with moderate sensitivity but are unlikely to provide the means to measure low abundance synaptic proteins since their signals will be quite low relative to the entire neuronal proteome. Second, these approaches may also suffer from the fact that many proteins localize to synapses in addition to other cellular locations. For example, many synaptic proteins are processed through the ER, Golgi apparatus, endosomal vesicles, and even lysosomes [39]. This situation is even more of a concern when investigating neurological disorders and diseases since non-synaptic pathogenic proteins accumulate or are removed from synaptic compartments in the context of disease [40,41]. For example, the loss of dopaminergic presynaptic terminals in PD brains is likely to have severe direct and compensatory effects on basal ganglia synaptic proteomes [42]. Collectively, the interpretation of “synaptic” proteomic datasets need to be interpreted with care and confirmed with additional experiments whenever possible. One interesting strategy that has provided an understanding of synapse type protein interaction networks has been the selective expression of tagged synaptic protein with affinity purification and MS analysis [43,44].

**Figure 3:**
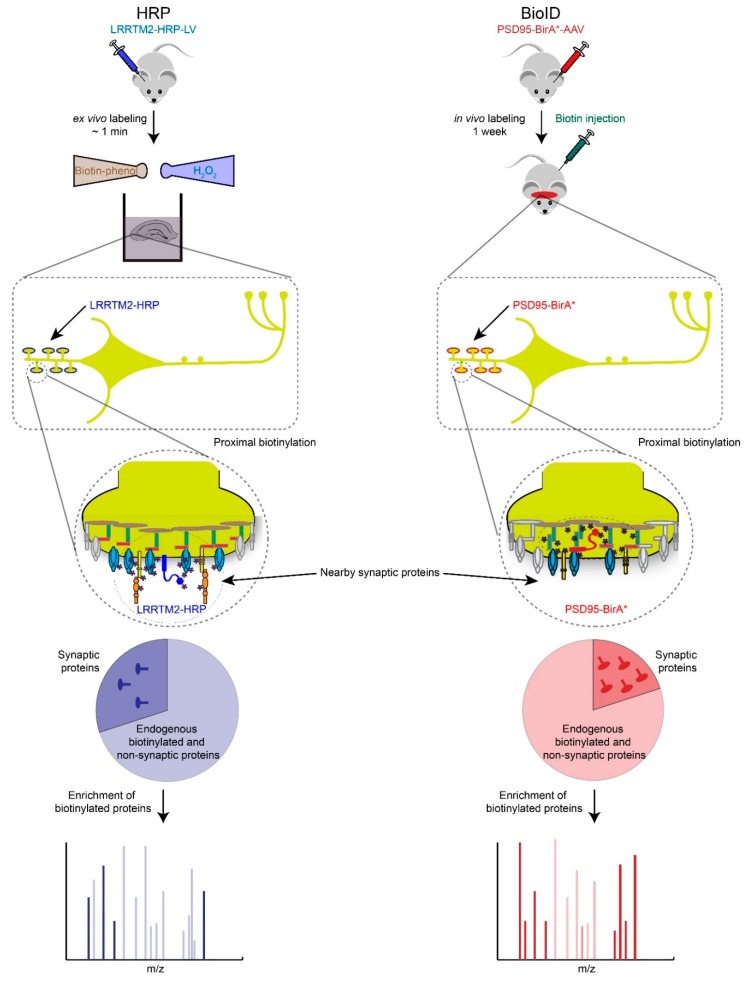
Proximity biotin-tagging strategies. Ascorbate or Horseradish Peroxidase (APEX or HRP)-based strategies are most suitable to profile synaptic proteomes in vitro or ex vivo because of biotin-phenol and H_2_O_2_ are toxic. Application of APEX or HRP based-strategies have been primarily used in cultured cells and neurons but we speculate here how these strategies could be used ex vivo. BioID-based proteomics works well in vivo but requires long incubation period (hours to days) to obtain high levels of biotinylated proteins, which raises the background biotinylation levels and increases the number of potential false positives.

Recently, Ting and Roux have reported two breakthrough methods to identify and quantitate spatially restricted proteomes by promiscuously tagging proteins with biotin via ascorbate or horseradish peroxidase (i.e., APEX, HRP), and BioID respectively [45,46]. They are based on similar principles and workflow but use different biotin-tagging enzymes, modified peroxidases or *E. coli* biotin ligase (BirA-R118G, or BirA*). These enzymes have been fused to a wide-range of different subcellular targeting proteins and peptides in order to probe a panel of different subcellular specific localizations, including mitochondria and synapses [45,47,48]. These strategies are well suited to determining synapse-type specific proteomes in vitro and in vivo, by fusing HRP, APEX or BirA* to PSD95 (e.g., excitatory post-synapses) or gephyrin (e.g., inhibitory postsynapses). Then small molecules that trigger enzyme-catalyzed biotinylation of proximal endogenous proteins are administered to animals or living cells [49,50]. Subsequently, the biotinylated proteins are enriched with streptavidin or anti-biotin antibody conjugated beads, and their identities and levels are determined by MS (Figure 3). In this way, HRP-LRRTM2 was recently used to identify 199 glutamatergic and 42 GABAergic synaptic cleft proteins in cultured neurons, and BioID revealed an elaborate inhibitory postsynaptic protein network in the developing mouse cortex [48,51]. Compared to traditional affinity purifications in combination with MS-based protein identification and quantification, proximity tagging technologies represent complementary, and in some cases more sensitive, analysis workflows that allow the measurement of low abundant proteins [44,52]. One key advantage of HRP/APEX and BioID over traditional affinity purification-based strategies is that the biotin protein tagging occurs in situ, which eliminates the requirement that protein-protein interactions survive detergent based lysis and biochemical purification.

However, there are two major disadvantages of proximity tagging technologies worth noting. First, just like BOSs, expression of promiscuous biotin-tagging enzymes may alter synaptic function. Second, a small panel of high abundance proteins are robustly biotinylated (i.e., pyruvate carboxylase, propionyl-CoA carboxylase, acetyl-CoA carboxylase) and tend to dominate the MS spectra and thus limit the detection of low abundance tagged proteins of interest. We have found that extensive peptide fractionation, rather than depleting the endogenously biotinylated proteins, is an effective work around. Finally, both HRP/APEX and BioID require well-designed bioinformatic strategies to identify top protein candidates from potentially long lists [47].

## 5. Conclusions and Future Perspectives

The recent development of highly sensitive and robust RNA sequencing (RNA-Seq) technologies has led to a boom in single cell analyses and significantly increased our understanding of global gene expression patterns of discrete neuronal populations in healthy and stressed conditions [53,54,55]. However, single cell RNA-Seq does not provide the subcellular resolution or protein expression data needed to interrogate discrete synapses or provide insight into the actual synapse-type specific protein expression patterns. MS-based strategies are beginning to bridge this gap, and over the coming years, we will eventually reach a deep understanding of neuron-type and synapse-type specific proteomes. Besides the ‘averaging problem’, many other obstacles are hindering our understanding of synaptic proteomes. Post translation modification (PTM) is one such example. In many conditions, the initiation of long-term potentiation requires SUMOylation and phosphorylation of many pre- and postsynaptic proteins [56,57,58]. PTMs complicate peptide spectra and consequently reduce the depth of traditional proteomic analyses. Now researchers are developing new sample preparation approaches and MS search algorithms (e.g., open search) to better handle PTMs [59]. Altogether, the accumulating knowledge of neuron-type specific and synapse-type specific proteomes will accelerate our basic understanding of synapses and neuronal networks and may pave a path towards the effective treatment of neurological disorders and diseases.
